# Screening for Posttraumatic Stress Disorder among Somali ex-combatants: A validation study

**DOI:** 10.1186/1752-1505-1-10

**Published:** 2007-09-06

**Authors:** Michael Odenwald, Birke Lingenfelder, Maggie Schauer, Frank Neuner, Brigitte Rockstroh, Harald Hinkel, Thomas Elbert

**Affiliations:** 1 University of Konstanz, Department of Psychology, Fach D25, 78457 Konstanz, Germany; 2 vivo international, Ancona, Italy; 3 GTZ International Services, Addis Abbeba, Ethiopia, and The World Bank MDRP (Multi-Country Demobilization and Reintegration Program of the Greater Great Lakes Region in Africa), Goma, Democratic Republic of Congo

## Abstract

**Background:**

In Somalia, a large number of active and former combatants are affected by psychological problems such as Posttraumatic Stress Disorder (PTSD). This disorder impairs their ability to re-integrate into civilian life. However, many screening instruments for Posttraumatic Stress Disorder used in post-conflict settings have limited validity. Here we report on development and validation of a screening tool for PTSD in Somali language with a sample of ex-combatants.

**Methods:**

We adapted the Posttraumatic Diagnostic Scale (PDS) to reflect linguistic and cultural differences within the Somali community so that local interviewers could be trained to administer the scale. For validation purposes, a randomly selected group of 135 Somali ex-combatants was screened by trained local interviewers; 64 of them were then re-assessed by trained clinical psychologists using the Composite International Diagnostic Interview (CIDI) and the Self-Report Questionnaire (SRQ-20).

**Results:**

The screening instrument showed good internal consistency (Cronbach's α = .86), convergent validity with the CIDI (sensitivity = .90; specificity = .90) as well as concurrent validity: positive cases showed higher SRQ-20 scores, higher prevalence of psychotic symptoms, and higher levels of intake of the local stimulant drug khat. Compared to a single cut-off score, the multi-criteria scoring, in keeping with the DSM-IV, produced more diagnostic specificity.

**Conclusion:**

The results provide evidence that our screening instrument is a reliable and valid method to detect PTSD among Somali ex-combatants. A future Disarmament, Demobilization and Reintegration Program in Somalia is recommended to screen for PTSD in order to identify ex-combatants with special psycho-social needs.

## Background

Like many other low-income countries, Somalia is challenged by the adverse effects of war and natural disasters with severe consequences for the mental health of its inhabitants. We recently showed that many Somalis, especially former Somali liberation fighters, who had fought against the Siad Barre regime, are currently functionally impaired by psychiatric disorders [1]. Here we report on a study conducted within the pilot Demobilization and Reintegration Program (DRP) Somalia that was financed by the European Community and implemented by the German Technical Cooperation (GTZ). This study aimed to develop a reliable and valid screening tool for Posttraumatic Stress Disorder (PTSD) among Somali ex-combatants.

Posttraumatic Stress Disorder (PTSD) represents a common, if not the most prevalent, mental health problem in community studies in post-conflict areas [2,3]. Elevated PTSD rates are particularly common in vulnerable groups suffering from multiple or continuous trauma [3-6], such as former child soldiers or tortured refugees [7-9]. Studies in western countries showed that PTSD frequently impairs the ability of former combatants to re-adjust to civilian life [10,11].

The use of assessment tools in post-conflict regions is difficult due to the lack of reliable and valid instruments [12]. In their review of studies on mental health and trauma in refugees, Hollifield and colleagues [13] concluded that the instruments used in most studies have had limited or untested reliability and validity for the specific population being studied. Coyne and colleagues [14] warn that the use of instruments with unknown validity in post-conflict settings might lead to inaccurate information about the mental health of individuals in those areas and might even lead to erroneous decisions concerning the distribution of scarce resources.

The authors of the Harvard Trauma Questionnaire (HTQ; [15]), an instrument designed for the assessment of PTSD symptoms frequently used in community-based studies in post-conflict areas, suggest that studies should always include a validation sub-study in order to define the appropriate population cut-off score [16] and avoid high numbers of misdiagnosed individuals – a recommendation which is often neglected.

The present study aimed to develop a short psycho-diagnostic screening instrument to assess for PTSD in Somalia, which is appropriate for the specific characteristics of the target population (e.g. high rate of illiteracy, low level of familiarity with questionnaires, Islamic background) and which can be administered by trained local interviewers in their native language. To develop this screening instrument, we translated and modified a widely used self-report instrument: the Posttraumatic Diagnostic Scale (PDS, [17]) into an 'assisted self-report' format (Somali-PDS). 'Assisted self report' means that the interviewer helps to fill in the questionnaire using a defined standard procedure, but does not probe or further inquire as in a clinical interview. Secondly, we aimed to determine convergent validity by comparing the results with DSM diagnoses based on expert interviews using the Composite International Diagnostic Interview (CIDI, [18]) and concurrent validity by comparison with other measures of psychopathology and drug intake. Our third goal was to compare the accuracy of two methods of defining positive screening cases, the original multi-criteria scoring method of the PDS and a single cut-off-score based on symptom items only, which is the most frequently used scoring method in community-based screening studies [19].

## Methods

### Study design

Participants of the screening interview were selected from a sample of 666 previously identified and electronically registered ex-combatants on the government payroll of the Republic of Somaliland (North-Western Somalia^1^) and selected to be participants of the Pilot Demobilization and Reintegration Program (DRP). Out of this group, 195 people were randomly selected and asked to participate in an interview, i.e. the screening for PTSD (from now on referred to as 'screening interview'). Despite the fact that they were on the government payroll, 47 could not be tracked due to their nomadic life style and lack of permanent residence. In addition, one person had died, four had moved to another town or country, and two were imprisoned. The remaining 141 ex-combatants were contacted by project staff, informed about the intention and procedure of the assessment, and invited to take part in the screening interview. Four individuals refused to participate. Of the remaining 137 participants, 2 did not complete the screening interview (response rate 135 of 141, i.e. 95.7%). The remaining 135 subjects were screened for PTSD symptoms by trained local staff (all non-experts). Additional topics of the screening interview consisted of demographic and clinical data such as the consumption of the stimulant drug khat. At the Horn of Africa and the neighboring regions, khat is a traditionally consumed substance with amphetamine-like properties [20,21], which is not illegal.

The first 64 interviewees of the screening interview were asked to participate in a second assessment conducted 2 to 14 days later. 62 of them completed this interview (response rate 96.9%). This assessment included a structured clinical interview conducted by a team of international researchers and clinicians who specialize in trauma. Trained interpreters assisted with these interviews. This second interview will be referred to below as the 'validation interview'. All interviewers and interpreters of the validation interview were blind with respect to the outcome of the screening interview.

### Subjects

Of the 135 participants of the screening interview, 133 were men and two were women. Their ages ranged from 19 to 70 years. Participants were involved in three different sections of the Somaliland armed forces: army, police, and custodian corps (prison wards)^2^. All participants were former members of the 'Somali National Movement' (SNM) and were receiving a monthly salary from the armed forces at the time of the study.

From this sample, 62 men and the 2 women were selected for the validation interview. This sample did not differ from the 71 ex-combatants who only participated in the screening interview with respect to age (*M *= 34.0, *SD *= 9.5 years vs. *M *= 34.3, *SD *= 10.2; t = 0.190, df = 132, p = .849), body mass index (*M *= 19.2, *SD *= 2.6 vs. *M *= 19.2, *SD *= 3.0; t = -0,072, df = 129, p = .943), military branch (army: 51.6% vs. 43.7%, police: 25.0% vs. 32.4%, prison wards: 23.4% vs. 23.9%; χ^2 ^= 1.084, df = 2, p = .582), or on the average amount of money spent per day on khat in the week preceding the screening interview (*M *= 1.04, *SD *= 1.66 US$ vs. *M *= 0.66, *SD *= 0.99 US$; t = -1.507, df = 108, p = .135). Importantly, the sum score of the screening instrument (Somali-PDS) did not differ either (*M *= 11.0, *SD *= 9.9 vs. *M *= 10.3, *SD *= 9.2, t = -.456, df = 133, p = .649).

The validation interview revealed that the average age of the ex-combatants when they started to actively fight in the war was 18.6 years (range 11 to 32 years; *SD *= 5.3; n = 54). At the time of their first military operation 69% were 18 or younger, 43% were 16 years or younger and 30% 15 or younger. Thus, a large fraction of the sample comprised former child soldiers. At the time of the validation interview, ex-combatants had an average of 5.2 years of formal education (*SD *= 4.2); 53% of them were married and their household included on average of 8.7 persons (*SD *= 5.1).

### Screening Interview

The screening interview assessed for symptoms of PTSD using a modified version of the Posttraumatic Stress Diagnostic Scale (PDS; [17]). The scale had been adapted to the Somali language, culture, and Islamic religion (Somali-PDS) according to recommendations for cultural adaptation [22]. The PDS is a widely used self-report instrument for the assessment of PTSD according to the DSM-IV criteria with good psychometric properties and validity [23-25]. According to Foa [23], the instrument achieved a Cronbach's Alpha of .92, test-retest reliability of .83, and a kappa of .74 (compared to the SCID-PTSD module) in a sample of 248 treatment-seeking individuals. These results are similar to those derived from two samples of general psychiatric outpatients [25] and of battered women [24].

In the first part of the instrument, a list of potentially traumatic events is presented and the respondent is asked to mark those event types that he or she experienced during his or her life. The participant is then asked to briefly describe the worst of these events and to indicate whether or not he or she felt extreme anxiety or helplessness during the event.  In the second part of the screening interview, the 17 DSM symptoms of PTSD are assessed in reference to the worst event. Participants are asked to rate the frequency of each symptom for the past four weeks on a 4-point scale (0 '*not at all/only one time*' to 3 '*five or more times a week/almost always*'; a symptom is counted if a score of 1 or higher is selected), as well as to indicate how long they have been experiencing these symptoms and how soon the symptoms began following the event. The next segment assesses difficulties in everyday functioning related to these symptoms. The scoring method established by Edna Foa is based on DSM-IV criteria for PTSD and was applied in this study: A positive screening case must fulfill all seven criteria indicated in the DSM-IV. The screening interview also included the assessment of demographic information (name, age, gender, military branch) and khat consumption (average money spent daily on khat during the last week).

Because many of the participants were illiterate, the self-report scoring of the PDS was adapted to an 'assisted self-report'. All items and answer categories were read to respondents by the interviewer. The interviewers marked the answers on the form without further probing. If a respondent indicated that he or she did not understand the meaning of the item the interviewer repeated the exact wording. If the respondent was not able to provide an answer the interviewer assisted by offering an alternative wording of the item without actively inquiring or probing. Interviewers received extensive training for this procedure.

### Validation Interview

The validation interview used the Composite International Diagnostic Interview for the DSM-IV (CIDI; WHO, 1997). This included the PTSD module (section K), and 13 items of the schizophrenia module (section G; G1, G2, G4, G6, G10, G14, G17, G18, G19, G20, G21) – the latter because psychotic symptoms are frequently co-morbid in veterans with PTSD [26] and their development was related to excessive khat chewing [1,27]. The CIDI has already been used in cross-cultural studies [28] and its excellent psychometric properties have been reported [29,30]. The former DSM-III-R PTSD module has been criticized for being less sensitive in detecting disorders [31] and has been extensively modified to meet the DSM-IV criteria [18]. Other studies criticized the strict skipping rules [32]. Based on these criticisms, clinicians in our study were instructed to ask and probe all items of the PTSD module and all selected items of the schizophrenia module. Psychotic symptoms were only included if the symptom was not related to dissociative phenomena or flashbacks.

In addition to PTSD and psychotic phenomena, symptoms of anxiety and depression were measured using the Self-Report Questionnaire-20 (SRQ-20; [33,34]). Items were read to the participant and the interviewer recorded the answers. The validity of answers to SRQ items were examined by probing questions [35].

In order to assess for exposure to traumatic events, a standard list, which asked for 15 situations with high face validity for the Somali military context, was used ('yes-no' format): fighting in combat (reported by 82%), witnessing combat (76%), killing or wounding enemies in combat (48%), being confronted with dead bodies in combat (88%), experiencing a life threatening accident or explosion (35%), witnessing serious accident or explosion (53%), suffering an injury by weapon (56%), witnessing injury by weapon (83%), witnessing violent death of relative or friend (52%), witnessing murder not in combat (35%), experiencing severe beatings or torture (26%), witnessing beatings or torture (21%), experiencing violent confiscation of property by officials (32%), experiencing harassment by armed personnel (36%), experiencing imprisonment (62%). The internal consistency of this list was satisfactory (Cronbach's α = .76).

Socio-demographic information and minor physical symptoms in the preceding month (cough, diarrhea, fever, hyperventilation, constipation, other; Cronbach's α = .67) were also assessed in the validation interview. Because it is well documented that ex-combatants with PTSD abuse psychotropic drugs more frequently compared to the ones without PTSD [36] khat consumption was quantified by items that already proved to be valid in field studies [1]. We assessed the money spent on khat in the week prior to the interview and the average time spent chewing khat per day. We also assessed the average number of cigarettes consumed per day as khat consumers usually smoke when chewing [37]. The average number of hours of sleep per day in the previous week was also assessed as patients with PTSD often have sleep difficulties [38,39].

### Cultural adaptation and translation of the PDS

The translation of the PDS to the Somali language as well as its cultural adaptation was carried out by groups of local bilingual and international experts, all of whom had received education in trauma-related concepts. In addition, group discussions and consultations with external specialists were dedicated to culturally specific meanings of items and typical experiences in Somalia. For example, we found no adequate Somali terms for the concepts 'stress' and 'trauma' and needed to circumscribe the meaning or find similar words. For example we translated 'stressful event' as 'difficult event', or 'traumatic event' as 'fearful incident' or 'reliving the traumatic event' as 'behaving as if you are once again in the situation that has caused you fear'. As a result, items such as those concerning rape and sexual experiences were modified to meet cultural and religious requirements. We could not ask *directly *about sexual abuse and violent sexual experiences, but had to design a hierarchical set of consecutive questions. Due to cultural and religious restrictions, we assessed only for rape and did not inquire into sexual contacts and molestation during childhood. Each subsequent question was asked only if the answer to the question before was positive. First, we asked whether a respondent had ever heard about a rape, then whether he had witnessed a rape. The next question would have been whether he knew the victim, and lastly whether he himself was the victim. The process of translation included a back translation, which was performed by independent professional translators. Items that were judged to be problematic were subjected to extensive discussions and retranslations, and were independently discussed with a second group of local staff. The process of back translation occurred as many times as necessary until all items had a clear and correct meaning.

### Training of local interviewers

Six local interviewers underwent a 10-day training by international researchers and clinicians. The training included theoretical education and practical exercises. Additionally, during the first two interviews they were directly supervised. Subsequently, team supervision was continuously provided throughout the four weeks of the screening exercise. The interpreters participated in the same theoretical education as interviewers, and translated and discussed all items of the CIDI and the structured clinical interview with expert team members as part of their training.

### Interview procedure

The screening interview lasted 20–40 minutes. All interviews took place in the Somalia Demobilization and Reintegration Program center in February and March 2002. Prior to the screening interview, local interviewers read a standardized explanation of the procedure to the participants, answered remaining questions, and asked them to sign an informed consent form. An additional informed consent was obtained for the validation interview, which was accomplished with the help of trained local interpreters and lasted approximately two hours.

### Ethical approval

The design, procedures and psycho-diagnostic instruments of the study were ethically approved by the Somaliland National Demobilization Commission (NDC) and the Ministry of Resettlement and Rehabilitation, Government of Somaliland, as well as by the German Technical Cooperation (GTZ).

### Data Analysis

Reliability of the Somali-PDS was evaluated using Cronbach's α (internal consistency). Convergent validity of the screening outcome was evaluated using kappa and coefficients of sensitivity and specificity. Receiver-operator curve (ROC) analysis was conducted to examine the diagnostic utility of the screening instrument compared to the CIDI [40]. Group differences were confirmed by student's t-Test (or Wilcoxon's test when not applicable) and *Chi*^2 ^test (or when appropriate Fisher's test). The data was analyzed using SPSS, version 11 for Macintosh.

## Results

### Screening reliability

For the sample of 135 participants, the internal consistency of the 17 symptom items of the Somali-PDS was high (Cronbach's α = .86). The corrected item total correlations for the 17 symptom items ranged between *r *= .33 (Item 11: feeling emotionally numb) and *r *= .61 (Item 4: feeling emotionally upset when reminded of the traumatic event), with a median of *r *= .47. In contrast to the symptom list, the internal consistency of the ten items of the event scale of the Somali-PDS was moderate (Cronbach's α = .54). The (corrected) item total correlations ranged between *r *= .12 (event 10: life-threatening illness) and *r *= .41 (Event 8: imprisonment). The median was *r *= .19.

### Convergent Validity

Table 1 compares the PTSD diagnosis based on the screening and the validation interviews, (62 subjects). The consistency of PTSD-diagnoses reached 90.3%, that is, in both interviews PTSD was diagnosed in nine subjects and in 47 it was not. Five subjects had a positive screening outcome, which was not confirmed by the validation interview (false positives). In one subject, the screening interview failed to detect PTSD. This confirmed a sensitivity of .90 and specificity of .90 (κ = .69, p < .001) for the screening interview with the Somali-PDS. In three of the five false positive cases, there was an overestimation of avoidance symptoms.

**Table 1:** Comparison of screening and validation interview. Comparison of the criteria for PTSD in the screening and validation interview. The two-way table shows the numbers of respondents with and without PTSD (rows; validation interview) and the positive and negative screening cases (columns).

		**Screening**		**Sum**
			
		**positive**	**negative**	
**Diagnosis based on expert Interview**	**PTSD**	9	1	10
	**No PTSD**	5	47	52
	**Sum**	14	48	62

Sensitivity .90

Specificity .90

Kappa = .69 (p < .001)

Fischer's exact test: p < .001

**Figure 1:**
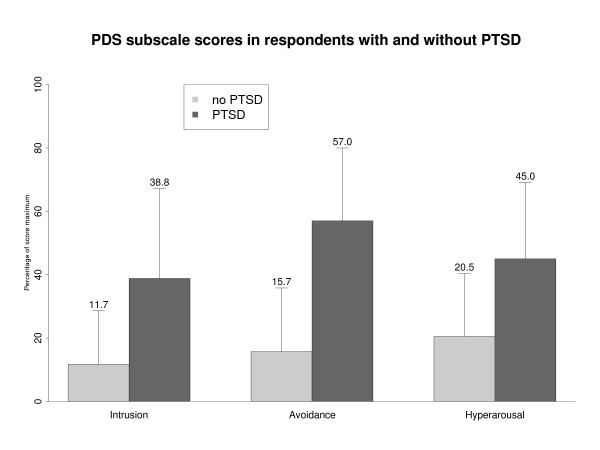
**Group differences**. Group differences between respondents with and without PTSD according to the CIDI: Group differences in sub-scales of the screening instrument between ex-combatants with PTSD (N = 10; dark-grey bars) and without PTSD (N =  52; light grey bars) in the clinical interview. Bars represent means of percentages of score maximum and standard deviation.

In the validation interview, respondents with PTSD (*n *= 10) showed higher symptom scores in the screening instrument compared to ex-combatants without PTSD (*n *= 52) with respect to the total symptom score as well as on the subscales level (see Figure 1; sum score: *M *= 23.2, *SD *= 10.0 vs. *M *= 8.7, *SD *= 8.0; *t *= -5.059, df = 60, *p *< .001; intrusions: *M *= 6.1, *SD *= 4.4 vs. *M *= 1.8, *SD *= 2.6; *z *= -3.269, *p *= .001; avoidance: *M *= 8.8, *SD *= 4.8 vs. *M *= 4.5, *SD *= 4.2; *t *= -2.926, df = 60, *p *= .005; arousal: *M *= 8.3, *SD *= 3.5 vs. *M *= 2.4, *SD *= 3.0; *t *= -5.452, df = 60, *p *< .001; functioning: *M *= 4.8, *SD *= 2.5 vs. *M *= 1.8, *SD *= 2.1; *t *= -4.0, df = 60, *p *< .001). At the item level, the largest differences between subgroups were found for item 16 ('*being overly alert, for example, checking to see who is around you, always being suspicious about what is going on behind you, etc.'*, *M *= 2.10, *SD *= 1.29 vs. *M *= .56, *SD *= 1.13; *t *= -3.9, df = 60, *p *< .001) and item 2 ('*having bad dreams or nightmares about the traumatic event'*: *M *= 1.20, *SD *= 1.32 vs. *M *= .65, *SD *= 1.10; *z *= -2.7; *p *= .006), in contrast to the smallest difference for item 6 ('*trying not to think about, talk about or have feelings about the traumatic event'*, *M *= 1.50, *SD *= 1.18 vs. *M *= 1.29, *SD *= 1.45, *z *= -.805; *p *= .421).

### Concurrent validity of the screening method

Ex-combatants with a positive screening outcome (PTSD) reported more anxiety and depression-related symptoms according to the SRQ-20, more psychotic symptoms (hallucinations and delusions according to the CIDI), more minor physical problems in the last month according to our symptom checklist and 126 minutes less sleep per 24 hours (see Table 2). Furthermore, there was a tendency toward more traumatic experiences according to our event list, although they did not report having been involved in armed conflicts longer than their comrades with a negative screening outcome. On average, ex-combatants with a positive screening outcome spent 2 hours per day more chewing khat, although the quantity of khat use (measured by the monetary value of the consumed khat) and the number of cigarettes were not significantly higher.

**Table 2:** Group differences. Differences between positive (PTSD) and negative screening cases (without PTSD). Differences between these two groups on measures which were assessed in the validation interview are shown. Means and standard deviations (in brackets) or percentages and numbers (in brackets) are reported.

	**Positive screening (14)**	**negative screening (48)**	**test statistic^1 ^(df)**	**p**
SRQ-20 sum score	9.07 (5.05)	2.52 (4.11)	-5.010 (62)	< .001
Psychotic symptoms	57% (8)	12% (6)	^6^	.001
Number of traumatic events	8.29 (3.00)	6.54 (2.98)	-1.94 (62)	.057
Years in armed conflict^2^	5.4 (4.0)	4.8 (4.6)	-.450 (59)	.655
Sum of minor physical symptoms in last month	1.71 (1.68)	.82 (1.21)	-2.24 (62)	.029
Average hours of sleep per 24 h in previous week	6.43 (2.95)	8.54 (2.39)	2.776 (62)	.007
Money spent on khat in last week (US$)^3^	7.38 (10.13)	3.05 (5.80)	-1.31^7^	.191
Average hours chewing khat per day in last week^3^	5.54 (5.94)	3.14 (2.46)	-2.14 (56)	.037
Khat used in combat ^4^	85% (11)	68% (27)	^6^	.305
Average numbers of cigarettes per day in last week ^5^	7.14 (7.52)	4.94 (6.94)	-1.027 (60)	.309

^1 ^student's t-test and Chi^2 ^were used; Wilcoxon was performed when variances were unequal; exact test according to Fisher-Yates was performed when expected frequency/cell was below 5

^2 ^different N: with PTSD 14, without PTSD 47

^3 ^different N: with PTSD 13, without PTSD 45

^4 ^different N: with PTSD 13, without PTSD 40

^5 ^different N: with PTSD 14, without PTSD 48

^6 ^exact test according to Fisher

^7 ^Wilcoxon's test

### Cut-off score

**Figure 2:**
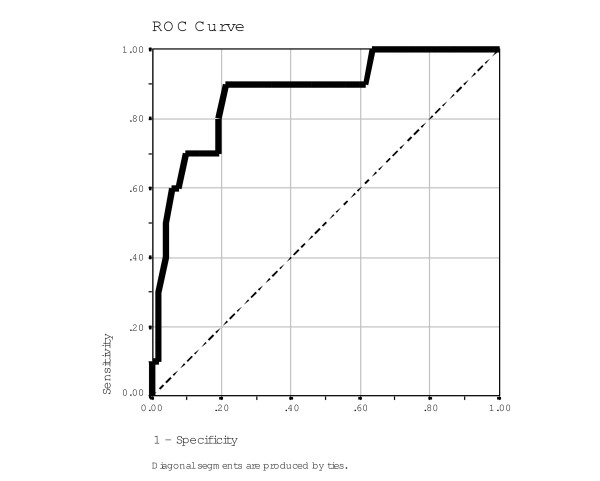
**Receiver-operator curve (ROC)**. Receiver-operator curve (ROC) showing sensitivity and 1-specificity of cut-off criteria based on the sum score of the screening instrument. Area under the curve = .874, SE = .062, p < .001. Sensitivity (.90) and specificity (.79) are highest with the cut-off criterion 13/14.

In most studies of post-war situations, PTSD cases are usually defined by a single cut-off score based on the sum score of symptom items only [41]. Here, we used the sum score of the 17 Somali-PDS symptom-items in order to create a cut-off score, and assessed its utility against the CIDI PTSD diagnosis by means of an ROC analysis. As illustrated in Figure 2, the area under the curve (AUC) indicates diagnostic accuracy better than chance of .874 (p < .001; SE = .062; CI 95% .752 to .996). Specificity and sensitivity clearly vary with the specific cut-off score chosen, with the highest magnitudes being achieved with a cut-off score of 13/14 (value "13" and lower are counted as negative and value "14" and higher as positive; sensitivity = .90, specificity = .79).

## Discussion

Extended screening of PTSD in countries affected by war and natural catastrophes is only feasible if local staff can be employed. The present study focused on the validity of a culturally adapted Somali version of Edna Foa's Posttraumatic Stress Diagnostic Scale ('Somali PDS'). In a sample of 135 mostly illiterate ex-combatants, Cronbach's Alpha reached .86. The comparison of screening with the 'Somali-PDS' and the PTSD diagnosis based on a standardized clinical interview (CIDI-K) in a sub-sample of 62 ex-combatants indicated a good convergent validity of the screening instrument (sensitivity = .90, specificity = .90). Additionally, the concurrent validity of the 'Somali-PDS' was supported by group differences in comorbid symptoms as assessed in the validation interview, e.g. more depressive and anxiety symptoms, more psychotic symptoms and more time per day spent on khat intake among ex-combatants with PTSD. The comparison between two scoring methods (cut-off score based on symptom severity score vs. multiple DSM IV criteria) revealed equal sensitivity but higher specificity with the multiple DSM IV criteria. In summary, this confirms the 'Somali PDS' is a valid screening instrument for ex-combatants in Somalia.

Consequences of war-related trauma cause enormous suffering and problems adjusting to post-war life in many parts of the world, with post-war Somalia being currently one of the most tragic cases. The international community has been waiting for years to implement a large DDR (Disarmament, Demobilization and Reintegration) program, but the continued upsurge of violence destroys these hopes. As soon as a peace accord is reached in Somalia, there will be 70,000 to 80,000 ex-combatants to be reintegrated into civilian society [42]. Based on our findings, we expect a high prevalence of PTSD among them. As PTSD is often followed by or comorbid with other problems, it constitutes a significant risk factor for reintegration failure. Our finding that individuals with PTSD report higher levels of minor physical symptoms, which is in line with other studies [43,44], might indicate that the general poor physical health of traumatized individuals affects their ability to be productive members of society. Particularly because of comorbid problems in the psychological domain, such as drug use or depression, and because of behavioral problems, PTSD should be recognized as an important factor in the reintegration process, as it is in western countries [36,45]. Also our findings suggest that PTSD among Somali ex-combatants is comorbid with higher levels of khat abuse, psychotic symptoms, anxiety and depression. Thus, future Somali DDR programs should be prepared to manage these associated symptoms through the development of adequate prevention and intervention tools. Fortunately, the inclusion of health and psycho-social issues in DDR programs is becoming an accepted standard of care [46] in recent years.

Conclusions from the present study are limited by sample size, which was relatively small for a validation study; thus, we think further studies on the reliability and validity of the Somali-PDS are needed. Furthermore, the sample group was not selected from the whole of the target population of ex-combatants, but rather was a selected group chosen to participate in the demobilization and reintegration program. The focus on ex-combatants might be questioned, as this group might not be representative of the general population, most of whom were refugees and have been victims of violence during wartime. In order to prove the validity of the instrument for other populations, we recently conducted a study to validate it within a civilian refugee population [47]. Furthermore, the use of the western concept of PTSD might be questioned. We believe that since scientific knowledge is generated mostly in the western world, it cannot simply be transferred to non-western cultures; but the responsibility to adapt these findings to and use them in the contexts where it is needed most should not be neglected [48]. Finally, in order to better understand the prevalence of war-related psychopathology in Somalia, the development of a more comprehensive measure including other types of psychological problems would have been helpful.

## Conclusion

In a first validation study with ex-combatants in Somaliland the 'Somali PDS', a newly developed screening instrument for PTSD, proved to be reliable and valid. We believe that this instrument will be helpful when the Somali DDR program is implemented as it identifies cases in need of special reintegration assistance.

## Competing interests

The author(s) declare that they have no competing interests.

## Authors' contributions

MO participated in the development of the study design, the selection of instruments, organized and carried out the training and supervision of local interviewers, conducted clinical interviews, performed the statistical analysis, was responsible for the interpretation of the data, drafted and revised the article.

BL assisted with the training and supervision of interviewers, carried out clinical interviews, assisted with the statistical analysis and took part in drafting and revision of the article.

MS participated in the development of the study design, the selection of instruments, carried out training of local interviewers, participated in the interpretation of the data and the revision of the manuscript.

FN participated in the development of the study design, the selection of instruments, and participated in the analysis and interpretation of the data. He took part in drafting and revision of the manuscript.

BR participated in the development of the study design, the selection of instruments, the interpretation of the data and the revision of the manuscript.

HH participated in the development of the study design, the training and supervision of local interviewers, the interpretation of the data and the revision of the manuscript.

TE participated in the development of the study design, the selection of instruments, trained and supervised local interviewers and conducted clinical interviews. He supervised the data analysis and took part in the interpretation of the data and the revision of the manuscript.

All authors read and approved the final version of the manuscript.

## Note

^1 ^This region corresponds to what had been the British protectorate Somaliland until 1960 and which unilaterally declared its independence in 1991 and established its own administration.

^2 ^After the end of the liberation war in 1991, the former members of the rebel army were detached to different branches of the new-built regular military forces.
